# Exercise training in the management of overweight and obesity in adults: Synthesis of the evidence and recommendations from the European Association for the Study of Obesity Physical Activity Working Group

**DOI:** 10.1111/obr.13273

**Published:** 2021-06-02

**Authors:** Jean‐Michel Oppert, Alice Bellicha, Marleen A. van Baak, Francesca Battista, Kristine Beaulieu, John E. Blundell, Eliana V. Carraça, Jorge Encantado, Andrea Ermolao, Adriyan Pramono, Nathalie Farpour‐Lambert, Euan Woodward, Dror Dicker, Luca Busetto

**Affiliations:** ^1^ Assistance Publique‐Hôpitaux de Paris (AP‐HP), Pitié‐Salpêtrière hospital, Department of Nutrition, Institute of Cardiometabolism and Nutrition Sorbonne University Paris France; ^2^ INSERM, Nutrition and Obesities: Systemic Approaches, NutriOmics Sorbonne University Paris France; ^3^ University Paris‐Est Créteil UFR SESS‐STAPS Créteil France; ^4^ Department of Human Biology, NUTRIM School of Nutrition and Translational Research in Metabolism Maastricht University Medical Centre+ Maastricht The Netherlands; ^5^ Sport and Exercise Medicine Division, Department of Medicine University of Padova Padova Italy; ^6^ Appetite Control and Energy Balance Group (ACEB), School of Psychology, Faculty of Medicine and Health University of Leeds Leeds UK; ^7^ Faculdade de Educação Física e Desporto CIDEFES, Universidade Lusófona de Humanidades e Tecnologias Lisbon Portugal; ^8^ APPsyCI—Applied Psychology Research Center Capabilities and Inclusion ISPA—University Institute Lisbon Portugal; ^9^ Obesity Management Task Force (OMTF) European Association for the Study of Obesity (EASO) Middlesex UK; ^10^ Obesity Prevention and Care Program Contrepoids; Service of Endocrinology, Diabetology, Nutrition and Patient Education, Department of Internal Medicine University Hospitals of Geneva and University of Geneva Geneva Switzerland; ^11^ Department of Internal Medicine D, Hasharon Hospital, Rabin Medical Center, Sackler School of Medicine Tel Aviv University Tel Aviv Israel; ^12^ Department of Medicine University of Padova Padova Italy

**Keywords:** exercise, obesity, physical activity, recommendations

## Abstract

There is a need for updated practice recommendations on exercise in the management of overweight and obesity in adults. We summarize the evidence provided by a series of seven systematic literature reviews performed by a group of experts from across Europe. The following recommendations with highest strength (Grade A) were derived. For loss in body weight, total fat, visceral fat, intra‐hepatic fat, and for improvement in blood pressure, an exercise training program based on aerobic exercise at moderate intensity is preferentially advised. Expected weight loss is however on average not more than 2 to 3 kg. For preservation of lean mass during weight loss, an exercise training program based on resistance training at moderate‐to‐high intensity is advised. For improvement in insulin sensitivity and for increasing cardiorespiratory fitness, any type of exercise training (aerobic, resistance, and combined aerobic or resistance) or high‐intensity interval training (after thorough assessment of cardiovascular risk and under supervision) can be advised. For increasing muscular fitness, an exercise training program based preferentially on resistance training alone or combined with aerobic training is advised. Other recommendations deal with the beneficial effects of exercise training programs on energy intake and appetite control, bariatric surgery outcomes, and quality of life and psychological outcomes in management of overweight and obesity.

## 1 INTRODUCTION

Physical activity is recognized as a “pillar” in the management of overweight and obesity, in parallel with dietary counseling, behavioral support, medication, and, in some instances, bariatric surgery.^1^ Physical activity is defined in broad terms as “any bodily movement produced by skeletal muscles that results in energy expenditure.”^2^ Exercise is viewed as a subcategory of physical activity that is “planned, structured, repeated with a given purpose, to maintain or increase physical fitness” (see Glossary, Table 1).^2^ Although the value of physical activity and exercise for maintaining health and preventing noncommunicable diseases is acknowledged as a public health “best buy,”^3^, ^4^ the role they may have for weight control remains debated both in the scientific^5^ and lay literature.^6^

**TABLE 1:** Glossary of terms (adapted from WHO Guidelines^3^ and PAGAC^4^)

Term	Definition
Physical activity	Any bodily movement produced by skeletal muscles that requires energy expenditure
Exercise training	Exercise is a subcategory of physical activity that is planned, structured, repetitive, and purposeful with primary purpose of improving or maintaining physical fitness, physical performance, or health.
Aerobic training	Programs based on forms of activities that are intense enough and performed long enough to maintain or improve an individual's cardiorespiratory fitness. Here, “aerobic” refers to moderate‐intensity aerobic training. On a scale relative to an individual's personal capacity, moderate‐intensity physical activity is usually a 5 or 6 on a scale of 0–10. Based on heart rate, moderate‐intensity physical activity is usually defined as 50%–70% of maximal heart rate.
Resistance training	Also referred to as “muscle‐strengthening activities”: programs based on activities that increase skeletal muscle strength, power, endurance, and mass and that involve major muscle groups (legs, back, abdomen, chest, shoulders, and arms). Intensity of resistance training is usually defined according to the one‐repetition maximum (1RM). Moderate intensity is usually defined as more than 60% of the 1RM.
High‐intensity interval training (HIIT)	Consists of short periods of high‐intensity anaerobic exercise, commonly less than 1 min, alternating with short periods of less intense recovery.
Physical fitness	A measure of the body's ability to function efficiently and effectively in daily‐life activities. Includes cardiorespiratory fitness, muscle strength, balance, and flexibility.

Several important reviews and position statements have been issued on the topic of physical activity and exercise regarding management of obesity during the 2000s.^7^, ^8^, ^9^ However, there has not been any systematic effort to get an overall update of more recent existing knowledge. Such overview would however be much needed to inform the design of practice guidelines for routine management of overweight and obesity in adults. In particular, there is a need for updated knowledge on the effects of various forms of exercise training programs (e.g., aerobic, resistance, or combined training) on weight loss, body composition changes with weight loss, and weight maintenance after weight loss in adults with overweight and obesity. Moreover, several topics of major importance have not been comprehensively addressed in previous reviews such as the effect of specific exercise training program in persons with overweight and obesity on intra‐hepatic fat, insulin sensitivity, blood pressure, cardiorespiratory and muscle fitness, eating behavior, hunger and satiety, and quality of life and psychological well‐being. To fill these gaps, a working group of European experts was convened in 2019 under the auspices of the European Association for the Study of Obesity (EASO^10^). EASO is a federation of professional membership associations from 36 countries across Europe and it produces guidelines as a key element of the education about obesity management.

The goals assigned to the EASO Physical Activity Working Group were to synthesize the literature on topics of importance in the field of exercise in management of overweight and obesity as published since 2010 and to write down the evidence on each of these topics in the form of systematic review papers, with meta‐analyses when applicable. The working group included clinical and nonclinical obesity experts with specific expertise in the field of physical activity and exercise: physiology, health care, psychology, and behavior change techniques. The literature search addressed the overall effect of exercise training on a series of outcomes of major interest in the management of overweight or obesity: body weight and body composition changes, metabolic health, physiological outcomes (fitness), behavioral outcomes (energy intake and appetite), bariatric surgery outcomes, and psychological outcomes. Attention was also directed to specific outcomes that had not been extensively reviewed before in the population of persons with overweight or obesity, such as the effects of exercise training on visceral and intra‐hepatic fat, muscular fitness, energy intake and appetite, health‐related quality of life, and the comparison between different exercise training modes.

The present paper summarizes the approach taken to synthesize recent literature on the above‐mentioned issues. The resulting evidence statements on the role of exercise in management of overweight and obesity are presented and discussed. This text therefore represents a summary of the material detailed in the accompanying papers on each outcome of interest. Recommendations regarding exercise in the management of overweight and obesity are presented as a final output of this work.

## 2 METHODS

All members of the working group followed the same methods to synthesize the evidence and format the recommendations, with variations as needed to reflect the evidence available in each field. The methodology followed a prespecified development process in three steps: (1) conducting of systematic reviews and meta‐analyses (SR‐MAs), (2) writing of evidence statements, and (3) designing of recommendations.

### 2.1 Systematic reviews and meta‐analyses

The systematic reviews followed the Preferred Reporting Items for Systematic Reviews and Meta‐Analysis (PRISMA) guidelines and were registered in the PROSPERO database (registration number CRD42019157823). Seven a priori defined research questions (Q1 to Q7) were addressed in the systematic reviews included in this supplement (Table 2). The details of methods used for each outcome under study are to be found in the corresponding papers of the series.

**TABLE 2:** List of research questions

Q1	Effect of exercise training interventions on weight loss, body composition changes and weight maintenance^11^
Q2	Effect of exercise training interventions on cardiometabolic health^12^
Q3	Effect of exercise training interventions on physical fitness^13^
Q4	Effect of exercise training interventions on energy intake and appetite^14^
Q5	Effect of exercise training interventions in the context of bariatric surgery^15^
Q6	Effect of exercise training interventions on quality of life and psychological outcomes^16^
Q7	Behavior change techniques to increase physical activity^17^

Briefly, depending on the topic, three to four electronic databases were searched (PubMed, Web of Science, Cochrane Library, EMBASE, PsychInfo, and SportDiscus) for original studies (Q2 to Q7) published up to 2020. The article addressing the first research question (Q1: effect of exercise on weight loss, body composition changes, and weight maintenance) was an overview of reviews, and the search was therefore limited to SR‐MAs. To avoid overlaps between SR‐MAs, we included only SR‐MAs published from January 2010 to December 2019.

Generic terms related to obesity and physical activity were used. Limits were set to include reviews/articles published in English. Reference lists from the resulting reviews and articles were also screened to identify additional articles. Articles were included if they involved adults (≥18 years including older adults) with overweight (body mass index, BMI ≥ 25 kg/m^2^) or obesity (BMI ≥ 30 kg/m^2^) as defined for Caucasian populations^18^ participating in an exercise training program. Presence of obesity comorbidities was not an exclusion criterion; for example, an article on subjects with obesity and type 2 diabetes was not excluded, whereas an article focused on subjects with type 2 diabetes (who usually have overweight or obesity, but this was not specified as an inclusion criterion) was excluded. Specifically, subjects with the following comorbidities were not excluded: type 2 diabetes, hypertension, dyslipidaemia, metabolic syndrome, liver disease (NAFLD/NASH), and osteoarthritis. Those with the following comorbidities were excluded: cardiovascular disease (coronary artery disease, stroke, and heart failure), cancers, rheumatoid arthritis, inflammatory bowel disease, kidney failure, neuropathy, severe orthopedic disorders (with important mobility limitations), intellectual deficiency, psychiatric conditions, fibromyalgia, asthma, and sleep disorders.

No minimum intervention length criterion was applied. Exercise training programs included sessions with one or more types of exercise (aerobic and/or resistance and/or high‐intensity interval training, HIIT). Exercise sessions could be fully supervised, partially supervised, or non‐supervised. Four systematic reviews (Q1, Q2, Q3, and Q5) included only randomized or non‐randomized controlled trials and three systematic reviews (Q4, Q6, and Q7) also included single‐group interventions. One review (Q1) that was an overview of reviews included only SR‐MAs of controlled trials. Exercise interventions in combination with other interventions (e.g., diet) with appropriate controls were included, except for Q3 that focused on exercise‐only interventions. Comparators included no intervention or usual care (i.e., intervention that any patient would have received in the framework of obesity management) or dietary interventions without exercise training or drug treatment.

Data were extracted using standardized forms. The effects of exercise were assessed using random‐effects meta‐analyses (Cochrane Review Manager 5.3 or Comprehensive Meta‐Analysis version 3). Effect sizes were reported as mean difference, MD, or standardized mean difference, SMD, alongside their 95% confidence intervals (CI) and *p* values and were categorized as large, medium, small, or negligible. A *p* value < 0.05 was considered statistically significant.

To assess study quality (good, fair, or poor), we used the tool developed by the National Heart, Lung and Blood Institute (NHLBI, USA) that has been previously used for defining guidelines for the management of obesity.^19^ The original assessment forms for SR‐MAs, controlled trials, cross‐over trials, and single‐group interventions were used. Publication bias was assessed by visual inspection of the funnel plots and when the number of included studies was >10, Egger's test and sometimes additional tests were performed (see individual papers for more details).

### 2.2 Evidence statements

Four to seven evidence statements were defined for each research question. The strength of each evidence statement was rated as high, moderate or low (Table S1) using the tool developed by the NHLBI.^19^ The strength of evidence represents the degree of certainty, based on the overall body of evidence, that an effect or association is correct.^19^

### 2.3 Recommendations

Recommendations were mainly formatted based on evidence statements with moderate to high strength of evidence. Members of the working group graded the recommendations as Strong Recommendation (Grade A), Moderate Recommendation (Grade B), Weak Recommendation (Grade C), Recommendation Against (Grade D), Expert Opinion (Grade E), or No Recommendation for or Against (Grade *N*) (Table S2).

## 3 RECOMMENDATIONS

A total of 15 recommendations are proposed regarding exercise training for (1) weight and fat loss, (2) weight maintenance after weight loss, (3) preservation of lean body mass during weight loss, (4) visceral fat loss and intra‐hepatic fat loss, (5) insulin sensitivity, (6) blood pressure, (7) cardiorespiratory fitness, (8) muscular fitness, (9) eating behavior, (10) hunger and satiety, (11) quality of life (physical component), (12) additional weight and fat loss with exercise after bariatric surgery, (13) physical fitness after bariatric surgery, (14) preservation of lean body mass after bariatric surgery, and (15) behavior change techniques for promoting physical activity (Table 3).

**TABLE 3:** Summary of recommendations

Recommendation	Grade	Corresponding ESs
Body weight and body composition		
1. Weight and fat loss		
Advise preferentially an exercise training program based on 150 to 200 min of aerobic exercise at least at moderate intensity.	A	1.1–1.2–1.4
Advise an exercise training program based on HIIT (i) only after thorough assessment of cardiovascular risk and (ii) with supervision.	B	1.3
Inform persons with overweight or obesity that expected weight loss is on average not more than 2 to 3 kg.	A	1.1–1.4
2. Weight maintenance after weight loss		
Advise a high volume of aerobic exercise (200 to 300 min/week of moderate‐intensity exercise).	E	1.7
3. Preservation of lean body mass during weight loss		
Advise an exercise training program based on resistance training at moderate‐to‐high intensity.	A	1.6
Cardiometabolic health		
4. Visceral fat loss and intra‐hepatic fat loss		
Advise preferentially an exercise training program based on aerobic exercise at moderate intensity.	A	1.5–2.4
Advise an exercise training program based on HIIT (i) only after thorough assessment of cardiovascular risk and (ii) with supervision.	B	1.5–2.4
5. Insulin sensitivity		
Advise any type of exercise training (aerobic, resistance, and combined aerobic or resistance) or HIIT (after thorough assessment of cardiovascular risk and under supervision).	A	2.1
6. Blood pressure		
Advise preferentially an exercise training program based on aerobic exercise at moderate intensity.	A	2.2–2.3
Physical fitness		
7. Cardiorespiratory fitness		
Advise any type of exercise training (aerobic, resistance, and combined aerobic or resistance) or HIIT (after thorough assessment of cardiovascular risk and under supervision).	A	3.1–3.2
8. Muscular fitness		
Advise an exercise training program based preferentially on resistance training alone or combined with aerobic training.	A	3.3–3.4
Energy intake and appetite		
9. Eating behavior		
Inform persons with overweight or obesity that an exercise training program will not have a substantial impact on energy intake but rather may improve eating behaviors.	B	4.1–4.4
10. Hunger and satiety		
Inform persons with overweight or obesity that exercise training may increase fasting hunger but improve the strength of satiety	B	4.2–4.3
Quality of life and psychological well‐being		
11. Quality of life (physical component)		
Advise an exercise training program based on either aerobic, resistance or a combination of both.	B	6.1
Bariatric surgery		
12. Additional weight and fat loss with exercise after surgery		
Advise an exercise training program based on a combination of aerobic and resistance training.	A	5.1
Inform that expected additional weight and fat loss is on average not more than 2 to 3 kg.	B	5.1
13. Physical fitness		
Advise an exercise training program based on a combination of aerobic and resistance training.	A	5.2
14. Lean body mass		
Advise an exercise training program based on a combination of aerobic and resistance training.	C	5.3
Behavior change techniques		
15. Habitual physical activity		
Preferentially use prompting behavioral practice and rehearsal in face‐to‐face behavior change interventions.	B	7.4

Abbreviations: ES, evidence statements; HIIT, high‐intensity interval training.

## 4 RESEARCH QUESTIONS AND CORRESPONDING EVIDENCE STATEMENTS

### 4.1 Q1—Weight loss, body composition changes, and weight maintenance

#### 4.1.1 Statement of the question

*In adults with overweight or obesity*

1‐a—What is the effect of exercise training programs on weight loss, changes in body composition (fat mass, visceral adipose tissue, and lean body mass), and weight maintenance?
1‐b—What are the effects of different types of exercise training (aerobic, resistance, aerobic and resistance combined, and HIIT) on these parameters?

#### 4.1.2 Search

Q1 was restricted to SR‐MAs published between 2010 and December 2019. The titles and abstracts of 3320 articles were screened against the inclusion and exclusion criteria, which resulted in 2337 articles being excluded and 123 being retrieved for full‐text review to further assess eligibility. Of the 123 articles, 12 SR‐MAs met the criteria and were included. A total of 149 unique original articles were included in the meta‐analyses. Three (25%) SR‐MAs were rated as good quality, 8 (67%) as fair quality, and 1 (8%) as poor quality. The most recent SR‐MA that focused on weight maintenance was published in 2014 (Johansson et al.^20^) and included only three original studies. Therefore, an additional search for original controlled trials on this outcome published between 2010 and July 2020 was performed. From 2422 articles identified and 13 articles retrieved for full‐text review, one controlled trial of fair quality was included.

#### 4.1.3 Evidence statements

Evidence statement 1.1: Aerobic training reduces body weight (by approximately 2 to 3 kg on average compared to controls without training and without dietary intervention and by 1 kg compared to resistance training alone) in groups of adults with overweight or obesity, independent of the duration of intervention.

➢*Strength of evidence: High*

Evidence statement 1.2: Aerobic training reduces body fat (by approximately 2 to 3 kg on average compared to controls without training and without dietary intervention and by 1 kg compared to resistance training alone) in groups of adults with overweight or obesity.

➢*Strength of evidence: Moderate*

Evidence statement 1.3: Aerobic training and HIIT lead to similar weight and fat loss in groups of adults with overweight or obesity, as long as the amount of energy expenditure is the same.

➢*Strength of evidence: Moderate*

Evidence statement 1.4: Aerobic training alone or combined with resistance training performed during a weight‐loss diet leads to an additional weight loss (of about 1.5 kg on average) and fat loss in groups of adults with overweight or obesity, compared to controls with diet only.

➢*Strength of evidence: High*

Evidence statement 1.5: Aerobic training and HIIT, but not resistance training, reduce abdominal visceral fat as measured by CT‐ or MRI‐scanning techniques in groups of adults with overweight or obesity, compared to controls without training.

➢*Strength of evidence: High*

Evidence statement 1.6: Resistance training, but not aerobic training, performed during a weight‐loss diet decreases the loss of lean body mass in groups of adults with overweight or obesity, compared to controls with diet only.

➢*Strength of evidence: Moderate*

Evidence statement 1.7: Adults who engage in large amounts of physical activity or aerobic exercise (≥250 min/week) are more likely to experience successful weight maintenance, according to retrospective analyses of randomized controlled trials (RCTs).

➢*Strength of evidence: Moderate*

### 4.2 Q2—Cardiometabolic health

#### 4.2.1 Statement of the question

*In adults with overweight or obesity*

2‐a—What is the effect of exercise training programs on insulin sensitivity, blood pressure, and intra‐hepatic fat?
2‐b—What are the effects of different types of exercise training (aerobic, resistance, aerobic and resistance combined, and HIIT) on these parameters?

#### 4.2.2 Search

The literature search for Q2 was limited to controlled trials published up to April 2020. Of the 6768 articles initially screened, 242 full‐text articles were assessed for eligibility and 54 met the inclusion/exclusion criteria and were included. The effect of exercise on insulin sensitivity, blood pressure, and intra‐hepatic fat was assessed in 36, 30, and 13 studies, respectively. In addition, the effect of exercise on insulin sensitivity and blood pressure was assessed according to the status of participants (with or without type 2 diabetes, with or without hypertension, respectively). Study quality was rated as good, fair, and poor in 11 (20%), 20 (37%), and 23 (43%) studies, respectively.

#### 4.2.3 Evidence statements

Evidence statement 2.1: Exercise training programs (aerobic, resistance, or HIIT) improve insulin sensitivity in groups of adults with overweight or obesity with or without type 2 diabetes.

➢*Strength of the evidence: High*

Evidence statement 2.2: Exercise training programs (aerobic, resistance, or HIIT) reduce systolic blood pressure by approximately 3 mmHg on average in groups of adults with overweight or obesity and with hypertension compared to controls without training.

➢*Strength of the evidence: High*

Evidence statement 2.3: Exercise training programs (aerobic, resistance, or HIIT) reduce diastolic blood pressure by approximately 2 mmHg on average in groups of adults with overweight or obesity with or without hypertension compared to controls without training.

➢*Strength of the evidence: High*

Evidence statement 2.4: Exercise training programs (aerobic, resistance, or HIIT) reduce intrahepatic fat in groups of adults with overweight or obesity compared to controls without training.

➢*Strength of the evidence: High*

### 4.3 Q3—Physical fitness

#### 4.3.1 Statement of the question

*In adults with overweight or obesity*

3‐a—What is the effect of exercise training programs on cardiorespiratory fitness and muscle strength?
3‐b—What are the effects of different types of exercise training (aerobic, resistance, aerobic and resistance combined, HIIT) on these parameters?

#### 4.3.2 Search

A systematic search of RCTs published up to December 2019 was performed. Of the 3068 articles initially screened, 162 full‐text articles were assessed for eligibility and 82 met the inclusion/exclusion criteria. Of these, 66 were included in the meta‐analyses. In these studies, comparisons were made either between an exercise group and a non‐exercise group, or between different types of exercise training. Study quality was rated as good, fair, and poor in 21 (32%), 28 (42%), and 17 (26%) studies, respectively.

#### 4.3.3 Evidence statements

Evidence statement 3.1: Aerobic, resistance, combined aerobic plus resistance, and HIIT interventions all increase VO_2_max compared with no exercise training in groups of adults with overweight or obesity.

➢*Strength of the evidence: High*

Evidence statement 3.2: HIIT interventions and interventions that include aerobic training are more effective in improving VO_2_max in groups of adults with overweight or obesity than resistance training alone.

➢*Strength of the evidence: Low*

Evidence statement 3.3: Resistance training interventions (resistance training alone or in combination with aerobic training) improves muscle strength compared with no exercise training in groups of adults with overweight or obesity.

➢*Strength of the evidence: High*

Evidence statement 3.4: Aerobic training interventions do not improve muscle strength in groups of adults with overweight or obesity.

➢*Strength of the evidence: Moderate*

### 4.4 Q4—Energy intake and appetite control

#### 4.4.1 Statement of the question

*In adults with overweight or obesity*

4‐a—What is the effect of exercise training programs on energy intake and appetite control (appetite ratings, eating behavior traits, and food reward)?
4‐b—What are the effects of different types of exercise training (aerobic, resistance, aerobic and resistance combined, and HIIT) on these parameters?

#### 4.4.2 Search

A systematic search of controlled trials, cross‐over trials, and single‐group interventions published up to October 2019 was performed. Only exercise training interventions were included as the combination with other interventions (e.g., diet and cognitive behavioral therapy) may influence energy intake and/or appetite control. Additionally, only exercise training interventions where diet was free to vary were included in the energy intake analysis. Comparators included no‐exercise controls. Of the 4593 articles initially screened, 155 full‐text articles were assessed for eligibility and 48 met the inclusion/exclusion criteria and were included. Study quality was rated as good, fair, and poor in 2 (4%), 7 (15%), and 39 (81%) studies, respectively.

#### 4.4.3 Evidence statements

Evidence statement 4.1: Exercise training does not increase average energy intake compared to the intake at baseline, nor is average energy intake substantially greater (~100 kcal) to that of non‐exercise controls in groups of adults with overweight or obesity.

➢*Strength of the evidence: Moderate*

Evidence statement 4.2: Exercise training leads to a small increase in fasting hunger compared to baseline, but there are no clear or consistent measurable effects on postprandial or daily hunger in groups of adults with overweight or obesity.

➢*Strength of the evidence: Moderate*

Evidence statement 4.3: Exercise training improves the strength of satiety compared to baseline in groups of adults with overweight or obesity.

➢*Strength of the evidence: Low*

Evidence statement 4.4: Exercise training leads to a small decrease in susceptibility to overconsumption through effects on behavioral traits and hedonic responses compared to baseline in groups of adults with overweight or obesity.

➢*Strength of the evidence: Moderate*

### 4.5 Q5—Bariatric surgery

#### 4.5.1 Statement of the question

*In adults with severe obesity undergoing bariatric surgery*

5‐a—What is the effect of preoperative exercise training programs on weight loss, changes in body composition, physical fitness, cardiometabolic health, habitual physical activity, and health‐related quality of life?
5‐b—What is the effect of post‐operative exercise training programs on weight loss, changes in body composition, physical fitness, cardiometabolic health, habitual physical activity, and health‐related quality of life?

#### 4.5.2 Search

Q5 was restricted to controlled trials published up to October 2019. Of the 2858 articles initially screened, 65 full‐text articles were assessed for eligibility and 31 met the inclusion/exclusion criteria and were included. A total of 22 distinct exercise training programs were analyzed, of which 18 programs were performed after bariatric surgery and 4 before surgery. The comparator was a group of adults undergoing bariatric surgery without exercise training. Study quality was rated as good, fair, and poor in 9 (43%), 4 (19%), and 8 (38%) studies.

#### 4.5.3 Evidence statements

Evidence statement 5.1: Exercise training (aerobic, resistance, or a combination of both) conducted after bariatric surgery results in an additional weight and fat loss (of 2.5 kg on average).

➢*Strength of the evidence: High*

Evidence statement 5.2: Exercise training (aerobic, resistance, or a combination of both) conducted after bariatric surgery improves cardiorespiratory fitness (VO_2_max, walking distance) and muscle strength.

➢*Strength of the evidence: High*

Evidence statement 5.3: Exercise training (aerobic, resistance, or a combination of both) conducted after bariatric surgery reduces the loss of lean body mass occurring during the first year after bariatric surgery, compared to controls without exercise after surgery.

➢*Strength of the evidence: Moderate*

Evidence statement 5.4: Aerobic training improves insulin sensitivity after bariatric surgery compared to controls without exercise after surgery.

➢*Strength of the evidence: Moderate*

Evidence statement 5.5: Exercise training (combination of aerobic and resistance) conducted before bariatric surgery may result in an additional weight loss after surgery and a larger increase in habitual physical activity.

➢*Strength of the evidence: Low*

### 4.6 Q6—Psychological outcomes and quality of life

#### 4.6.1 Statement of the question

*In adults with overweight or obesity*

6‐a—What is the effect of exercise training programs on quality of life, depression, anxiety, perceived stress, body image, and other psychological outcomes?
6‐b—What are the effects of different types of exercise training (aerobic, resistance, aerobic and resistance combined, and HIIT) on these parameters?

#### 4.6.2 Search

A systematic search of controlled trials and single‐group interventions published up to October 2019 was performed. Of the 1298 articles initially screened, 74 full‐text articles were assessed for eligibility and 36 met the inclusion/exclusion criteria. Twenty‐one studies were included in meta‐analysis. Most studies (32 out of 36) were RCTs. Study quality was rated as good, fair, and poor in 14 (39%), 14 (39%), and 8 (22%) studies, respectively. Supervised or semi‐supervised exercise interventions, assessing one or more psychosocial outcomes (both pre‐ and post‐exercise or compared with control), were included. Studies involving multicomponent interventions (e.g., exercise paired with a behavioral intervention or diet) were excluded if the isolated effect of exercise could not be determined (e.g., diet + exercise vs. control and behavioral intervention vs. control).

#### 4.6.3 Evidence statements

Evidence statement 6.1: Exercise training programs can increase quality of life's physical component in groups of adults with overweight or obesity.

➢*Strength of the evidence: Moderate*

Evidence statement 6.2: Exercise training programs appear to be able to increase vitality and mental health in groups of adults with overweight or obesity.

➢*Strength of the evidence: Low*

Evidence statement 6.3: Exercise training programs are not able to reduce depression‐related outcomes in groups of adults with overweight or obesity.

➢*Strength of the evidence: Moderate*

Evidence statement 6.4: Exercise training programs appear to improve self‐efficacy and autonomous motivations for exercise in groups of adults with overweight or obesity.

➢*Strength of the evidence: Low*

Evidence statement 6.5: Combined aerobic plus resistance exercise training programs appear to induce greater improvements in quality of life, compared to aerobic‐only or resistance‐only training programs, in adults with overweight or obesity.

➢*Strength of the evidence: Low*

### 4.7 Q7—Behavior change techniques

#### 4.7.1 Statement of the question

*In adults with overweight or obesity*

7‐a—What are the most effective behavior change techniques for increasing physical activity in face‐to‐face interventions?
7‐b—What are the most effective behavior change techniques for increasing physical activity in digital interventions?

#### 4.7.2 Search

The search for Q7 was restricted to RCTs published up to October 2019. Of the 1760 articles initially screened, 168 full‐text articles were assessed for eligibility and 53 met the inclusion/exclusion criteria and were included. From these, 35 studies referred to digital trials and 28 studies to face‐to‐face trials. Study quality was rated as good, fair, and poor in 15 (24%), 26 (42%), and 21 (34%) studies, respectively. No previous systematic review and meta‐analysis have examined the effectiveness of motivational behavior change techniques^21^ along with the behavior change technique taxonomy (BCTTv1),^22^ or separately analyzed behavior change techniques effectiveness in changing physical activity in digital and face‐to face interventions in adults with overweight or obesity. Behavior change interventions that primarily or secondarily aimed at increasing physical activity were included. Comparators included no intervention, standard care, or dietary intervention without a physical activity practice or counseling component.

#### 4.7.3 Evidence statements

Evidence statement 7.1: Effective behavior change techniques for increasing physical activity in digital behavior change interventions seem to differ from effective behavior change techniques in face‐to‐face interventions in groups of adults with overweight or obesity.

➢*Strength of the evidence: Low*

Evidence statement 7.2: Digital behavior change interventions using goal setting, social incentive and graded tasks might result in greater increases in physical activity than interventions that do not use these behavior change techniques, in groups of adults with overweight or obesity.

➢*Strength of the evidence: Low*

Evidence statement 7.3: Digital behavior change interventions using self‐monitoring of behavior might not result in increases in physical activity as high as those obtained with interventions that do not use this behavior change technique, in groups of adults with overweight or obesity.

➢*Strength of the evidence: Low*

Evidence statement 7.4: Face‐to‐face behavior change interventions (taking place physically, on site) prompting behavioral practice and rehearsal might lead to more favorable physical activity outcomes, compared to face‐to‐face interventions that do not use this behavior change technique, in groups of adults with overweight or obesity.

➢*Strength of the evidence: Moderate*

## 5 GAPS IN EVIDENCE AND PRIORITY RESEARCH NEEDS

In general, findings of the series of reviews performed show gaps in current knowledge in a number of aspects:

1. Interindividual variability in response to exercise and its consequences for management of persons with overweight or obesity needs further exploration.
2. Better understanding of the importance of exercise, and different types and timing of exercise, on appetite control and eating behavior would greatly improve management strategies.
3. The value of physical activity counseling versus structured exercise training should be better defined.
4. Effects of HIIT were found of interest on several outcomes; however, feasibility and acceptability in real‐life settings would need further delineation in persons with overweight or obesity.
5. More specific questions include defining the volume of physical activity required for weight maintenance after weight loss, including after bariatric surgery.
6. Optimal timing of exercise training interventions after bariatric surgery would require investigation.
7. Assessing whether the effects of aerobic and strength training in persons with overweight or obesity are of about the same magnitude as in lean subjects would help better tailor prescriptions.
8. More evidence would be needed on psychological outcomes (such as body image, anxiety, perceived stress, and life satisfaction) as well as on effects of individual versus group‐based exercise training.
9. Which combination of behavior change techniques (face‐to‐face or digital) are most effective for increasing physical activity and how they should be administered remains an open question.
10. Improved knowledge of dose–response relationships between volume of exercise and effects on given outcomes would be needed to design quantitative guidelines specific to persons with overweight or obesity.
11. The importance of reducing sedentary behavior (e.g., sitting time) should be assessed in management of overweight and obesity.
12. Capacity building of instructors and coaches in offering exercise programs adapted to the needs and capabilities of persons with obesity should be developed.
13. Finally, how to increase adherence to prescribed exercise, especially in the long term, should be explored in depth. The value of wearable devices and apps for this purpose needs to be examined in subjects with overweight or obesity.

## 6 IMPLICATIONS FOR CLINICAL PRACTICE

Based on the evidence gathered through our systematic search and analysis of the literature on exercise in the management of overweight and obesity in adults, some implications for practice can be proposed. It is important to emphasize the numerous health benefits to be gained with higher physical activity and fitness levels in persons with overweight or obesity. Given that effects on weight (and fat) loss as such were found of modest size, the implementation of exercise training programs in persons with overweight or obesity should primarily aim to increase physical fitness, reduce cardiometabolic risk, and improve quality of life. These benefits of exercise will very likely improve overall health, even without substantial change in body weight.

Within the scope of a comprehensive approach of management of overweight and obesity, exercise prescription will be carried out in conjunction with dietary advice, psychological interventions, pharmacotherapy when needed and/or available, and in persons with severe obesity, bariatric surgery.^19^, ^23^ The five A's strategy consisting Ask, Assess, Advise, Agree, and Assist (or Arrange)^24^, ^25^ appears well adapted in this perspective, especially for the aim to individually tailor the exercise prescription to the needs, preferences, capacity, corpulence, and health status of patients.

The topic of physical activity and exercise should be discussed as part of each encounter between a health professional and any patient with overweight or obesity (“Ask”). Information about the benefits expected should be provided. An evaluation of habitual physical activity and physical fitness is a logical follow‐up of dietary and lifestyle assessment in patients (“Assess”). Simple questionnaires designed for use in the setting of general practice can help.^26^ There is currently no specific recommendation about when to perform a maximal exercise test in subjects with overweight or obesity (without diabetes). Such testing may however be important to search for underlying coronary heart disease in high‐risk patients and/or to adapt the exercise load on a quantitative basis.^27^ A specific goal should be defined for the patient and specific activities or programs proposed to reach that goal (“Advise”). Goals will be shared between health professionals and patients (“Agree”). Counseling will be tailored to the individual needs of the patients taking into account physical fitness, co‐morbidities, stage of change regarding physical activity, barriers to increase physical activity, and opportunities offered in the living environment. The process of counseling will develop over time with frequent reassessment and subsequent adaptation (“Assist”). Interventions rest on behavior change and a major challenge is how to improve adherence to a new lifestyle over time.^28^

When recommending exercise for adults with overweight or obesity, it is important to balance any positive with potential negative effects on health. In the general population, exercise is associated with an increased risk of musculoskeletal injuries and adverse cardiac events, but there is evidence from non‐randomized trials and observational studies that the benefits of exercise far outweigh the risks in most adults.^29^ Musculoskeletal injuries are the most frequent negative side effects of exercise. There is however very little information on musculoskeletal injuries in adults with overweight or obesity during exercise interventions. Some studies in this setting did not find more injuries in the intervention group than in the control group,^30^, ^31^ while other studies reported more injuries in an exercise intervention group.^32^, ^33^ We are not aware of studies that directly compared the injury risk in adults with or without overweight or obesity. The incidence of both acute myocardial infarction and sudden death is greatest in the least habitually physically active individuals performing unaccustomed physical activity.^34^ It is likely that a larger percentage of adults with overweight or obesity falls in this inactive group compared to lean subjects. On the other hand, the largest benefits on all‐cause mortality are attained when this group is moved to an at least “moderately active” level.^35^ By analogy with the general population, overall it seems prudent to advise habitually inactive adults with obesity to become more active by a gradual progression of exercise volume by adjusting exercise duration, frequency, and/or intensity.^29^

## CONFLICT OF INTEREST

No conflict of interest statement.

## AUTHOR CONTRIBUTIONS

All authors participated to the writing of evidence statements and recommendations. JMO and AB drafted the manuscript, and authors critically revised the manuscript.

## Supporting information

**Table S1.** Rating the strength of the evidence^1^
**Table S2**. Rating the strength of the recommendations^1^
