# Supplementary material for: Exercise training in the management of overweight and obesity in adults: Synthesis of the evidence and recommendations from the European Association for the Study of Obesity Physical Activity Working Group
Source: Obes Rev. 2021 Jun 2;22(Suppl 4):e13273. doi: 10.1111/obr.13273 (PMC8365734; doi:10.1111/obr.13273)
Supplement: Supplementary file 1 — Table S1. Rating the strength of the evidence1 Table S2. Rating the strength of the recommendations1 [file OBR-22-e13273-s001.pdf]

SUPPORTING INFORMATION

Exercise training in the management of overweight and obesity in adults: synthesis of the evidence and recommendations from the EASO Physical Activity Working Group

Jean-Michel Oppert*[1], Alice Bellicha[2,3], Marleen A. van Baak[4], Francesca Battista[5], Kristine Beaulieu[6], John E. Blundell[6], Eliana V. Carraça[7], Jorge Encantado[8], Andrea Ermolao[5], Adriyan Pramono[4], Nathalie Farpour-Lambert[9,10], Euan Woodward[9], Dror Dicker[9,11], Luca Busetto[9,12]

***Corresponding Author**

Prof. Jean-Michel Oppert, MD PhD

Service de Nutrition, Hôpital Pitié-Salpêtrière

47-83 Boulevard de l'Hôpital

75013 PARIS, France

Tel: +33(1)42175782

Fax:+33(1)42175963

E-mail: jean-michel.oppert@aphp.fr

Table S1. Rating the strength of the evidence [1]

Quality rating	Type of evidence
**High**	• Well-designed, well-executed RCT that adequately represent populations to which the results are applied and directly assess effects on health outcomes.<br>• Meta-analyses of such studies.<br>➢ **Highly certain about the estimate of effect. Further research is unlikely to change our confidence in the estimate of effect.**
**Moderate**	• RCT with minor limitations affecting confidence in, or applicability of, the results.<br>• Well-designed, well-executed nonrandomized controlled studies and well-designed, well-executed observational studies.<br>• Meta-analyses of such studies.<br>➢ **Moderately certain about the estimate of effect. Further research may have an impact on our confidence in the estimate of effect and may change the estimate.**
**Low**	• RCT with major limitations.<br>• Nonrandomized controlled studies and observational studies with major limitations affecting confidence in, or applicability of, the results.<br>• Uncontrolled clinical observations without an appropriate comparison group (e.g., case series, case reports).<br>• Physiological studies in humans.<br>• Meta-analyses of such studies.<br>➢ **Low certainty about the estimate of effect. Further research is likely to have an impact on our confidence in the estimate of effect and is likely to change the estimate.**

Table S2. Rating the strength of the recommendations [1]

Grade	Strength of recommendation
A	**Strong recommendation**<br>➢ There is high certainty based on evidence that the net benefit is substantial
B	**Moderate recommendation**<br>➢ There is moderate certainty based on evidence that the net benefit is moderate to substantial, or there is high certainty that the net benefit is moderate
C	**Weak recommendation**<br>➢ There is at least moderate certainty based on evidence that there is a small net benefit
D	**Recommendation against**<br>➢ There is at least moderate certainty based on evidence that there is no net benefit or that risks/harms outweigh benefits.
E	**Expert opinion ("There is insufficient evidence or evidence is unclear or conflicting, but this is what the Work Group recommends.")**<br>➢ Net benefit is unclear. Balance of benefits and harms cannot be determined because of no evidence, insufficient evidence, unclear evidence, or conflicting evidence, but the Work Group thought it was important to provide clinical guidance and make a recommendation. Further research is recommended in this area.
N	**No recommendation for or against ("There is insufficient evidence or evidence is unclear or conflicting.")**<br>➢ Net benefit is unclear. Balance of benefits and harms cannot be determined because of no evidence, insufficient evidence, unclear evidence, or conflicting evidence, and the Work Group thought no recommendation should be made. Further research is recommended in this area.

Reference

Jensen MD, Ryan DH, Apovian CM, et al. 2013 AHA/ACC/TOS guideline for the management of overweight and obesity in adults: a report of the American College of Cardiology/American Heart Association Task Force on Practice Guidelines and The Obesity Society. Circulation. 2014;129(25 Suppl 2):S102-38. doi:10.1161/01.cir.0000437739.71477.ee
